# Synthesis and characterization of novel donor–acceptor type electrochromic polymers containing diketopyrrolopyrrole as an acceptor and propylenedioxythiophene or indacenodithiophene as a donor†

**DOI:** 10.1039/c8ra03570a

**Published:** 2018-06-26

**Authors:** Xinfeng Cheng, Xiuping Ju, Hongmei Du, Yan Zhang, Jinsheng Zhao, Yu Xie

**Affiliations:** College of Chemistry and Pharmaceutical Engineering, Nanyang Normal University Nanyang 473061 P. R. China; Shandong Key Laboratory of Chemical Energy Storage and Novel Cell Technology, Liaocheng University Liaocheng 252059 P. R. China j.s.zhao@163.com; Dongchang College, Liaocheng University Liaocheng 252059 P. R. China; College of Environment and Chemical Engineering, Nanchang Hangkong University Nanchang 330063 P. R. China xieyu_121@163.com

## Abstract

A range of low band gap donor–acceptor conjugated polymers (P1–P3) with backbones composed of diketopyrrolopyrrole (DPP), propylenedioxythiophene (ProDOT) and indacenodithiophene (IDT) units were designed and synthesized using the Stille coupling reaction. The optical, electrochemical and electrochromic properties of the resultant polymers were thoroughly characterized. These polymers showed exceptional solubility in common organic solvents and displayed thermal stability at a high temperature. The optical and electrochemical measurements revealed slight variations in the maximum absorptions and oxidation peaks depending on the intrinsic D–A ratio in each polymer, and narrow band gaps lower than 1.60 eV were found for these polymers. Upon oxidation, the polymer films exhibit distinct color changes (pale violet-red to dark gray for P1, rosy brown to silver for P2, atrovirens to light grey for P3) in the VIS and NIR regions. Moreover, the electrochromic switching studies indicated that these polymers have favorable switching properties, such as rapid response speed and high optical contrast and coloration efficiency, and are outstanding candidates for electrochromic applications.

## Abstract 2

Three novel D–A type conjugated copolymers were prepared, illustrating excellent electrochromic properties, such as desirable color switches, high optical contrasts, fast response time, and high color efficiency.

## Graphical abstract

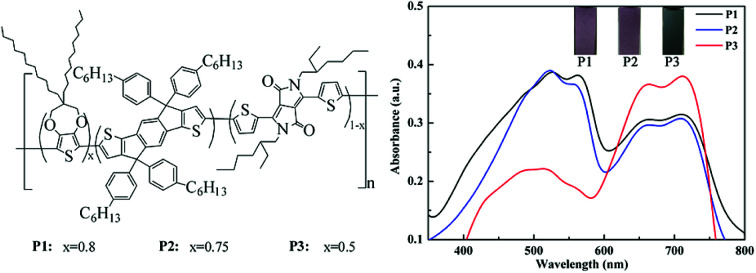

## 1. Introduction

Conjugated polymers (CPs) with promising electronic and optical properties find diverse applications in field effect transistors, solar cells, electrochromic (EC) devices, light-emitting diodes and sensors.^1–5^ In terms of their EC performance, these materials display multiple colorations, high optical contrasts, fast switching times and decreased power consumption during operation compared with inorganic electrochromes.^6,7^ The attractive optoelectronic properties and solution-processing performances of conjugated electrochromic polymers can be fine-tuned *via* chemical and structural modifications of their backbones or side chains.^8–12^ To this end, various methods have been developed. A well-established strategy is the donor–acceptor (D–A) approach, in which electron-donating and electron-accepting heterocycles alternated along the polymer chain to generate EC materials with a low band gap, fine-tuned optoelectronic characteristic and solution processability.^13–17^ The color changes of these D–A type polymers can be easily controlled through the facile manipulation of the positions and intensities of absorption peaks by regulating the D–A ratio as well as the chemical constitution of the polymers.^18–21^

Indaceno[1,2-*b*:5,6-*b*′]dithiophene (IDT), owning thiophene-based fused heteroacenes, has evolved into a versatile building block for organic optoelectronic materials, such as polymer photovoltaic cells and field-effect transistors.^22–26^ Owing to its larger coplanar fused structure, the degree of π electron delocalization is extended, endowing it with a more electron-rich structure, which can be served as a good electron donor. In addition, the solubility of the IDT-containing CPs can be optimized due to its ease of functionalization by attaching side alkyl chains at two sites.^27^ Therefore, incorporation of electron-donating IDT units into the EC polymers may achieve materials with improved electrochromic performances. For instance, Xu and coworkers synthesized a number of novel D–A type polymers containing IDT derivatives. These polymers exhibited high coloration efficiency, fast coloration times, desirable contrasts in both VIS and NIR regions, and reasonable optical memory and stability, making them a promising candidate for application in electrochromic displays.^26^

Particularly, the optical and electronic performances of many conjugated polymers containing a popular electron accepting unit, *i.e.* 1,4-diketopyrrolo[3,4-*c*]pyrroles (DPP), have attracted considerable research interests.^28–34^ Owning to its highly conjugated lactam structure of DPP molecule, π–π interactions and electron-withdrawing effects were strengthened,^35,36^ which allows the electrochromic polymers absorb intensely in the NIR region and ambipolar transport with improved hole and electron mobilities.^37^ Recent studies take more attention to use the DPP-based conjugated polymers as electrochromic materials with attractive electrochemical and electrochromic switching properties.^18,38–41^ Inspired with the unique electron-withdrawing characteristic of the DPP unit, to the best of our knowledge, the studies about electrochromism of IDT/DPP containing conjugated donor–acceptor polymers are scarce.

Propylenedioxythiophene (ProDOT) is one of the most promising derivatives of ethylenedioxythiophene (EDOT). Owing to the more electron-donating property of propylenedioxy bridge, the oxidation potential of ProDOT was dramatically decreased.^42–44^ Therefore, it has been emerging as another star comonomer used in preparing D–A type conjugated polymers with excellent optoelectronic performances and enhanced long-term stability.^45–48^ Herein, in this study, rational design of solution-processable IDT/ProDOT/DPP based D–A type copolymers was conducted by using alkylated IDT, ProDOT as donor units and thiophene-flanked DPP as acceptor unit. The optical and electrochemical properties of the resultant copolymers were investigated by using UV-Vis-NIR spectroscopy and cyclic voltammetry (CV). The effect of the D/A ratio in D–A copolymers on the EC performances consisting of optical contrast, coloration efficiency and switching stability were characterized in detail as presented below.

## 2. Experimental

### 2.1 Materials

Chloroform (99.0%), methanol (95%), diethyl ether (99%), ether (99%), acetone (99.5%), toluene (95%) and other solvents were all bought from Laiyang Fine Chemical Factory. Acetonitrile (HPLC grade) and dichloromethane (HPLC grade) were purchased from Aladdin chemicals and used directly without further purification. 1-Bromododecane, malonic acid diethyl ester, 3,4-dimethoxy-thiophene, sodium hydride, *p*-toluene-sulfonic acid (*p*TSA), *N*-bromosuccinimide (NBS, 99%), lithium aluminum hydride and tetrabutylammonium hexafluorophosphate (TBAPF_6_, 98%) were purchased from Aladdin chemicals. Bis(triphenylphosphine)dichloropalladium (Pd(PPh_3_)_2_Cl_2_), magnesium sulfate (MgSO_4_, ≥99.0%) and tetrahydrofuran (THF) were bought from Energy chemicals (Shanghai, China). THF was distilled over Na in the presence of benzophenone before use. 3,6-bis(5-bromothiophen-2-yl)-2-(2-ethylhexyl)-5-(2-methylhexyl)pyrrolo[3,4-*c*]pyrrole-1,4(2*H*,5*H*)-dione, (4,4,9,9-tetrakis(4-hexylphenyl)-4,9-dihydro-*s*-indaceno[1,2-*b*:5,6-*b*′]dithiophene-2,7-diyl)bis(trimethylstannane) were purchased from Derthon Optoelectronic Materials Science Technology Co., Ltd, (Shenzhen, China). Indium-tin-oxide (ITO) coated glass with sheet resistance of <10 Ω □^−1^ were bought from CSG Display Technologies, (Shenzhen, China). ITO glass was cleaned by ethanol, acetone and distilled water prior to use. The electrolyte is 0.2 M TBAPF_6_/ACN.

### 2.2 Instrumentation

^1^H and ^13^C NMR spectra were measured on a Varian AMX 400 spectrometer. FT-IR spectra were recorded on a NICOLET 6700 IR spectrometer. Gel permeation chromatography (GPC) analysis was performed on a Waters 2414 system with chloroform as eluent. The electrochemical characteristics were tested by cyclic voltammetry (CV) with CHI 760C electrochemical workstation connected by a three-electrode cell. The working, counter and reference electrodes were in sequence of ITO glass, platinum wire and silver wire. The silver wire was chosen as pseudo reference electrode, which was calibrated to be 0.02 V *vs.* SCE. Spectroelectrochemical and colorimetry measurements were carried out on a Varian Cary 5000 spectrophotometer using a UV-cuvette as electrolytic cell with the above three-electrode system. Scanning electron microscope (SEM) images were obtained by using a Hitachi SU-70 thermionic field emission SEM. Digital photographs were taken by a Nikon D610 camera. The polymer films obtained by spray-casting method were adopted by using an airbrush to spray the prepared polymer solution in chloroform on the ITO conducting glass.

### 2.3 Synthesis

#### 2.3.1 Synthesis of monomers

**Scheme 1:**
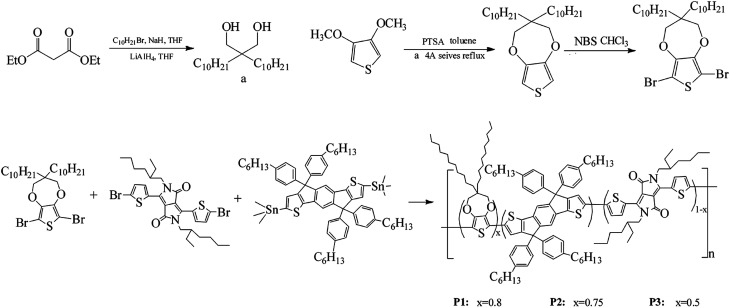
Synthetic routes of monomers and polymers (P1–P3).

The synthesis process of 6,8-dibromo-3,3-bis-decyl-3,4-dihydro-2*H*-thieno[3,4-*b*][1,4]dioxepine (Monomer a) was showed in Scheme 1. In a dried three-neck flask, 200 mL of dry THF, 3.5 mol of 1-bromododecane and 3.5 mol of sodium hydride were added and degassed with argon. Upon further degassed and then cooled to 0 °C, 1.15 mol diethyl malonate was added dropwise, and keep stirring and refluxing overnight. Subsequently, the solution was poured into 200 mL of brine and extracted 2 times by using ether, and then removed the alkyl bromide and solvent under reduced pressure. The remaining product was added dropwise at 0 °C to the same flask where 200 mL of dry ethyl ether and 2 mol of LiAlH_4_ powder were already joined, and then stirred under argon atmosphere for 20 h at room temperature to give 2,2-bisdecyl-1,3-propanediol intermediate (yield: 82%). Afterwards, 0.1 mol of the obtained diol, 0.05 mol of 3,4-dimethoxythiophene and 0.05 of mol *p*-TSA were combined in 200 mL of toluene in a 500 mL flask connected by a Soxhlet extractor with 4 Å molecular sieves in the thimble. The mixture was refluxed for 24 hours at 120 °C, and then cooled and washed by distilled water. Then the residue was purified by column chromatography on silica gel with 5 : 1 hexane/DCM to give a liquid 3,3-bis-decyl-3,4-dihydro-2*H*-thieno[3,4-*b*][1,4]dioxepine (yield: 78%). ^1^H NMR (CDCl_3_, 400 MHz, ppm): *δ* = 6.42 (s, 2H), 3.84 (s, 4H), 1.26 (m, 36H), 0.88 (t, 6H). ^13^C NMR (CDCl_3_, 101 MHz, ppm): *δ* = 149.69, 104.63, 77.53, 77.00, 76.68, 43.72, 29.63, 29.33, 22.68, 14.13 (see Fig. S1†).

Then 3,3-bis-decyl-3,4-dihydro-2*H*-thieno[3,4-*b*][1,4]dioxepine was brominated to give the final product, 6,8-dibromo-3,3-bis-decyl-3,4-dihydro-2*H*-thieno[3,4-*b*][1,4]dioxepine (Monomer a).^49^ 5 g of 3,3-bis-decyl-3,4-dihydro-2*H*-thieno[3,4-*b*][1,4]dioxepine and 5.5 g of NBS were dissolved with 130 mL of chloroform in a 200 mL flask, and then stirred at room temperature in the dark for 24 hours. Then the mixture was purified by column chromatography on silica gel with 5 : 1 hexanes/methylene chloride to give a final transparent oil of Monomer a (yield: 86%). The overall yield of Monomer a was up to 55%. ^1^H NMR (CDCl_3_, 400 MHz, ppm): *δ* = 3.90 (s, 4H), 1.26 (m, 36H), 0.88 (t, 6H). ^13^C NMR (CDCl_3_, 101 MHz, ppm): *δ* = 147.14, 90.63, 77.98, 77.00, 76.68, 43.99, 31.65, 29.72, 22.69, 14.10 (see Fig. S2†).

#### 2.3.2 Synthesis of polymers

The polymers were synthesized by Stille coupling reaction of 6,8-dibromo-3,3-bis-decyl-3,4-dihydro-2*H*-thieno[3,4-*b*][1,4]dioxepine (Monomer a),3,6-bis(5-bromothiophen-2-yl)-2-(2-ethylhexyl)-5-(2-methylhexyl)pyrrolo[3,4-*c*]pyrrole-1,4(2*H*,5*H*)-dione (Monomer b), (4,4,9,9-tetrakis(4-hexylphenyl)-4,9-dihydro-*s*-indaceno[1,2-*b*:5,6-*b*′]dithiophene-2,7-diyl)bis(trimethylstannane) (Monomer c). Briefly, 0.3079 g Monomer a, 0.0881 g Monomer b and 0.8000 g Monomer c were dissolved in 120 mL toluene, followed by addition of 0.0205 g Pd(PPh_3_)_2_Cl_2_. Then the mixture was stirred magnetically and refluxed for 48 hours in argon atmosphere at 125 °C. Afterwards, the solvent toluene was removed under vacuum, and the residue was redissolved in small amount of chloroform. Then the crude products were precipitated in 120 mL methanol from chloroform. After filtration, the precipitates were extracted by Soxhlet extractor using methanol and acetone as solvent for 12 hours. After a subsequent vacuum drying of the residue at 35 °C, a palevioletred solid was obtained for polymer P1 with a yield of 73%.

With the same method, polymer P2 and P3 were also synthesized. The respective feed ratios of P2 and P3 were shown in Scheme 1.

#### P1

^1^H NMR (CDCl_3_, 400 MHz, ppm): *δ* 8.942–8.823 (s, 3H), 7.486–6.883 (m, 150H), 4.146–3.803 (s, 20H), 2.701–2.421 (m, 53H), 1.669–1.021 (m, 372H), 0.968–0.785 (s, 116H), 0.406–0.245 (s, 7H), 0.105–0 (s, 5H). Yield: 73%.

#### P2

^1^H NMR (CDCl_3_, 400 MHz, ppm): *δ* 8.950–8.810 (s, 3H), 7.460–6.951 (m, 107H), 4.153–3.797 (s, 16H), 2.642–2.416 (s, 35H), 1.646–1.074 (m, 238H), 0.999–0.710 (s, 77H), 0.341–0.230 (s, 2H), 0.101–0 (s, 8H). Yield: 72%.

#### P3

^1^H NMR (CDCl_3_, 400 MHz, ppm): *δ* 9.413–8.972 (s, 21H), 7.666–6.870 (m, 58H), 5.285–4.967 (s, 8H), 2.996–2.514 (d, 26H), 2.144–0.784 (m, 162H), 0.220–0 (d, 16H). Yield: 76%.

## 3. Results and discussion

### 3.1 Characterization of the synthesized polymers

**Fig. 1:**
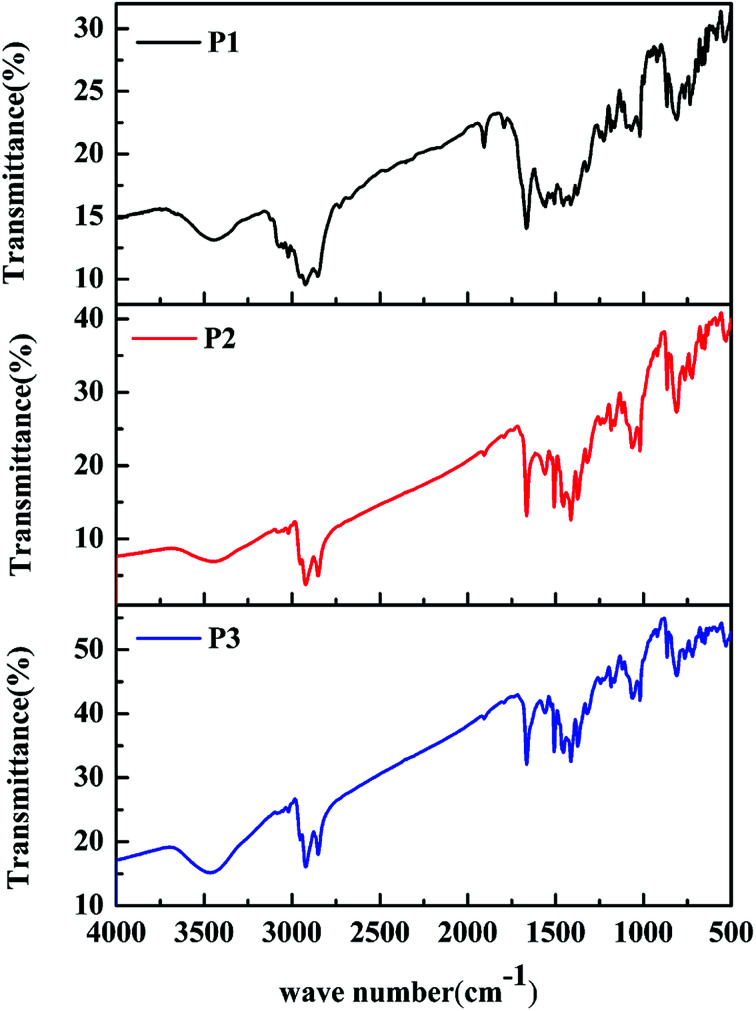
FT-IR spectra of polymers P1–P3.

The classic Stille coupling reaction was adopted to synthesize the polymers P1–P3, as shown in Scheme 1. The polymers were characterized by FT-IR spectra. As shown in Fig. 1. The absorption peaks at about 869, 813 and 721 cm^−1^ in the fingerprint region were owing to the bending vibration of polysubstituted benzene ring from IDT units. The absorption bands at about 1374, 1317 and 1507 cm^−1^ were due to the C–C, C–S and C=C stretching vibration, respectively. The characteristic bands at 1182 and 1560 cm^−1^ were originated from the C–N and C=O stretching vibration of DPP units. Additionally, the bands at 1065 and 1664 cm^−1^ were contributed to the stretching vibration of C–O from ProDOT units and benzene ring from IDT units, respectively. Meanwhile, the peaks at about 2850, 2921 and 3465 cm^−1^ were assigned to the saturated and unsaturated C–H stretching vibration, respectively. From the above results, the characteristic absorptions originated from the ProDOT, IDT and DPP units can be clearly found in the spectra of the polymers, indicating that the ProDOT/IDT/DPP terpolymers were successfully synthesized. Besides, the FT-IR spectra of the three polymers are similar to each other, due to their similar polymer structures obtained from the same monomers. Only the difference in absorption intensity is attributed to their different monomer ratios. In addition, the chemical structures of these polymers were further verified by ^1^H NMR analyses (see Fig. S3–S5†). Moreover, GPC data of the three polymers demonstrated that the weight-average molecular weight (*M*_w_) and poly dispersity (PDI) of the polymers were 17 600 (PDI ∼ 1.69) for P1, 19 300 (PDI ∼ 1.75) for P2, and 15 200 (PDI ∼ 1.88) for P3, respectively (see Fig. S6†).

### 3.2 Electrochemical behavior of polymers

**Fig. 2:**
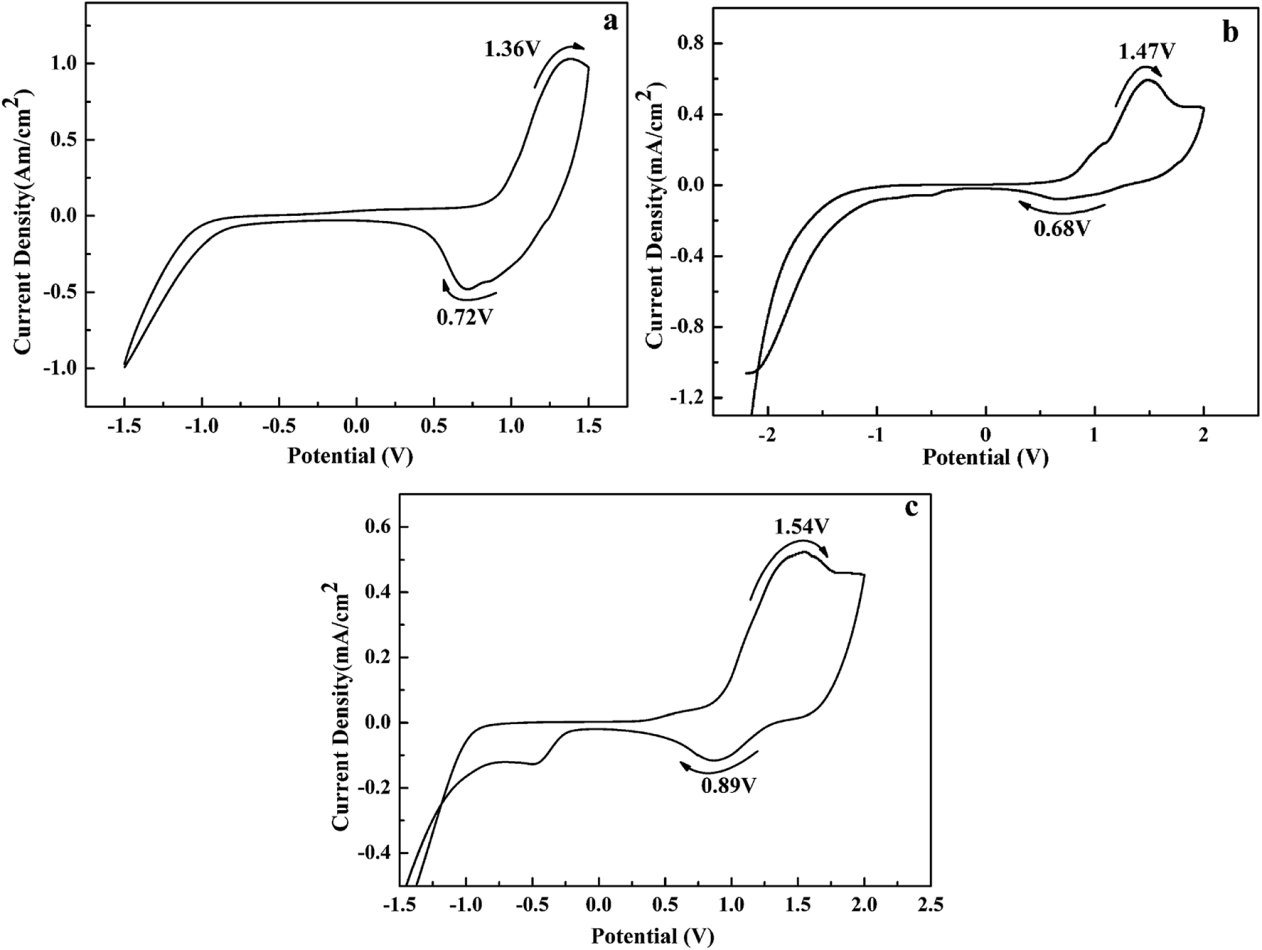
CVs of the polymeric film coated on ITO glass: (a)P1; (b)P2; (c)P3.

The electrochemical properties of the polymers were studied by cyclic voltammetry (CV) in 0.2 M TBAPF_6_/ACN as a supporting electrolyte. As shown in Fig. 2, the redox couples occur at 1.36 and 0.72 V for P1, 1.47 and 0.68 V for P2, 1.54 and 0.89 V for P3, corresponding to the p-doping/p-dedoping process of the polymers. Additionally, the onset oxidation potential (*E*_onset_) of P1–P3 were determinated to be 0.72, 0.73 and 0.75 V, respectively. There is a slight increase of *E*_onset_ values from P1 to P3, due to that the electron donor units in the polymers backbone is decreased gradually from P1 to P3, which can make the copolymers easier to be oxidated.^50^ Besides, there are no n-doping process observed in the CVs in the negative potential region. All the oxidation potentials of the polymers were less than 1.6 V at a low level, which is crucial for a successful electrochromic material. Additionally, it was found that these polymers could retain 82–87% of their original electroactivity after 500 cycles, indicating that these polymers have reasonable redox stability (see Fig. S7†).

### 3.3 Morphology

The surface and bulk morphologies of the polymeric films were tested by SEM analyses. The morphology of P1 film displayed a homogeneous permutation superficial structure, and there were some little particles bump closely packed in the surface of the film (see Fig. S8†). The main function of this granuliform structure is possibly to promote the doping anions to move into or out of the polymer backbone in the doping and de-doping processes. Additionally, the morphologies of P2 and P3 films have some similarities with P1 (see Fig. S8†), maybe owing to the reason that P1–P3 polymers were composed of the same electron donor and acceptor units.

### 3.4 Spectroelectrochemical properties of polymers

**Fig. 3:**
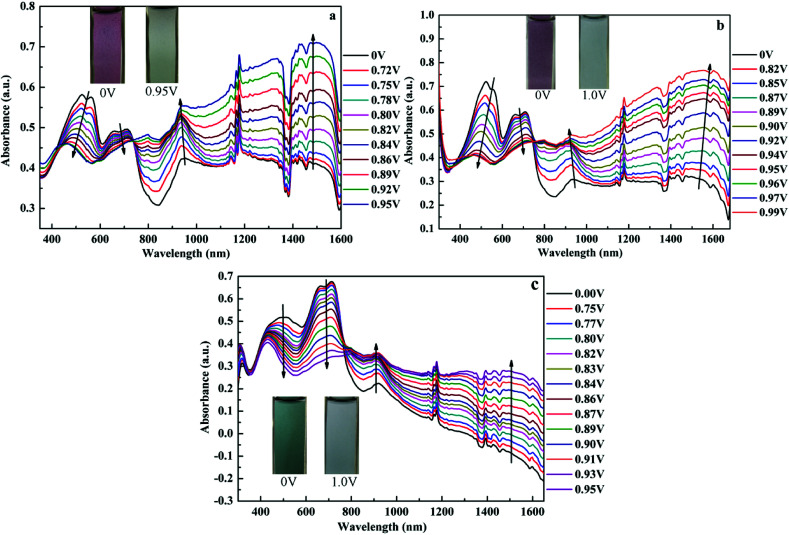
UV-Vis-NIR spectra of P1 (a), P2 (b) and P3 (c) at various potentials. The insets show the color changes between neutral and oxidized states.

Spectroelectrochemistry plays an important role in the characterization of polymers to describe the absorption spectra changes and the electronic properties. Spectroelectrochemistry of the polymeric films spray-coated onto ITO glass was investigated in 0.2 M TBAPF_6_/ACN solution. As shown in Fig. 3, in the neutral state (at 0 V), there appeared dual-band absorption spectra in the VIS region located at 524 and 712 nm for P1 (Fig. 3a), which is ascribed to π–π* electron transition. When the potential increasing from 0 V to 0.95 V for P1, the π–π* transition peaks decreased and other two bands appeared and intensified at 934 and 1483 nm in the NIR region owing to the formation of polarons and bipolarons.^51^ There was a distinct color change from palevioletred (0 V) to darkgray (0.95 V) observed for P1 with the increase of potential. Similar absorption changes in the UV-vis and NIR regions were observed for P2 and P3 film when varying potential from 0 to 0.95 V (or 0.99 V) (Fig. 3b and c), which was associated with an analogical color variation from rosybrown to silver relative for P1, and atrovirens to lightgrey for P3. Moreover, the optical behaviors upon oxidation also exhibit the isosbestic points at 735 nm for P1, 748 nm for P2, 766 nm for P3, implying that they were successfully interconverted between the neutral state and the doped state.

### 3.5 Optical properties of polymers

**Fig. 4:**
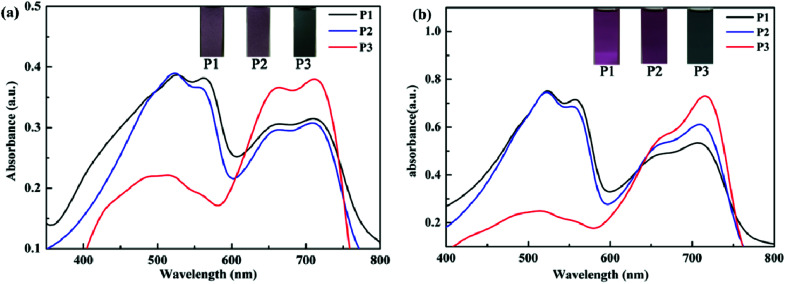
The absorption spectra and their photos of P1–P3 in the form of film (a) and solution (b).

The UV-Vis absorption spectra of the polymers in film and solution states were tested in neutral states. As seen in Fig. 4, P1–P3 displayed obvious dual-band absorption not only in the solution but also in the form of film. In the solid film state, the absorption peaks occurred at 524 nm for P1, 522 nm for P2, 711 nm for P3. The solutions of P1–P3 showed the maximum absorptions at 522 nm for P1, 522 nm for P2, 715 nm for P3, respectively. There exists a distinct red shift for P3 as compared with P1 and P2, because of their better planar and stiff backbone which is beneficial for charge transfer in spite of the minimum donor–acceptor ratio. Comparing the maximum absorption potential of polymers in the form of film and solution, there exists no significant difference. The reason maybe that the π–π stacking in the film can enhance the interchain interaction and lower the absorption energy, but the strong steric hindrance effect can adversely weaken the π–π stacking, as a result the difference closed. Besides, the insets also clearly show color changes of P1–P3 in neutral states. For P1, the film is palevioletred, the solution is violet. For P2, the film is rosybrown, the solution is orchid. For P3, the film is atrovirens, the solution is darkslategray.

According to the CV curves and spectroelectrochemical spectra, we can get some parameters, including onset oxidation potential (*E*_onset_), onset of the optical absorption spectra in neutral states (*λ*_onset_), maximum absorption wavelength in solution (*λ*_max,solution_) and film (*λ*_max,film_). Based on *E*_onset_ and *λ*_onset_, we can calculate optical band gap (*E*_g_) and HOMO/LUMO energy levels, which are calculated according to the following equations:^52,53^*E*_g_ = 1240/*λ*_onset_,*E*_HOMO_ = −e(*E*_onset_ + 4.0 + 0.04)*E*_LUMO_ = *E*_HOMO_ + *E*_g_

The corresponding data are tabulated in Table 1. Here, 0.04 is a correction parameter because the Ag reference electrode used here was not a standard electrode. The band gap (*E*_g_) of P1–P3 is 1.44, 1.46 and 1.48 eV respectively. These polymers have a low level band gaps because of the polymerization, and also have an increasing tendency from P1–P3. This phenomenon is owing to the effect of donor–acceptor ratios, *i.e.*, there exists decreased electron donor content from P1–P3, which is beneficial to reducing the band gaps.

**Table 1:** Onset of the optical absorption wavelength in neutral states (*λ*_onset_), maximum absorption wavelength in solution (*λ*_max,solution_) and film (*λ*_max,film_), onset oxidation potential (*E*_onset_), optical band gap (*E*_g_), HOMO/LUMO energy levels of all copolymers

Copolymers	*λ*_onset_(film), nm	*λ*_max_(solution), nm	*λ*_max_(film), nm	*E*_onset_, V	*E*_g_, eV	HOMO, eV	LUMO, eV
P1	864	522	524	0.72	1.44	−4.76	−3.32
P2	847	522	522	0.73	1.46	−4.77	−3.31
P3	837	715	711	0.75	1.48	−4.79	−3.31

### 3.6 Electrochromic switching studies

**Fig. 5:**
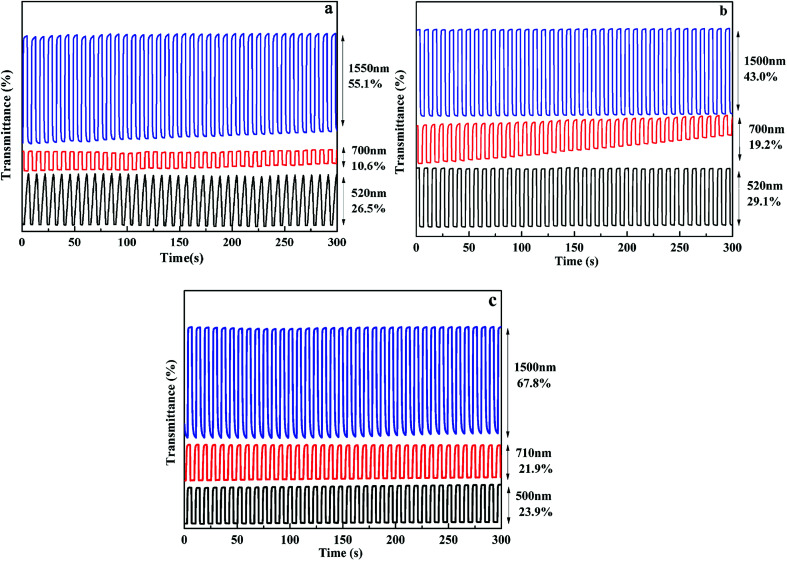
Optical transmittance change of P1(a), P2(b), P3(c) at different wavelengths.

In order to investigate the electrochromic switching property of the polymer films, the optical contrast (Δ*T*%) was tested with multi-potential steps technique, as shown in Fig. 5. When the square wave potential with an interval of 4 s was applied to the films, the polymer films exhibit high contrast ratios both in the VIS and NIR regions. As for P1 film, when voltage varied between 0 V and 0.95 V, the optical contrast is 26.5% at 520 nm, 10.6% at 700 nm in the VIS region and 55.1% at 1550 nm in the NIR region. For P2 film, the optical contrast is 29.1% at 520 nm, 19.2% at 700 nm in the VIS region and 43.0% at 1500 nm in the NIR region. While for P3 film, the optical contrast is 23.9% at 500 nm, 21.9% at 710 nm in the VIS region and 67.8% at 1500 nm in the NIR region. The results demonstrate that all polymers have a larger optical contrast in the NIR region as compared to that in the VIS region, informing that these electrochromic materials have better utilization potentiality in the NIR region. In addition, the optical contrast of them remained 78–96% of their initial values after 500 cycles at full switching potentials between redox states accompanied by unperturbed color change, informing that these polymers have favorable optical reversibility (see Fig. S9†).

Response time (*t*_95%_), which is defined as necessary time for 95% of the full optical switch when tuning between neutral state and doped state, is determined and summarized in Table 2. The response time of P1 is 1.74 s at 520 nm, 0.37 s at 700 nm and 0.78 s at 1550 nm. For P2, the response time is 0.42 s at 520 nm, 0.30 s at 700 nm and 0.60 s at 1550 nm. While for P3, the response time is 0.57 s at 500 nm, 0.18 s at 710 nm, 1.50 s at 1500 nm. All of them showed fast response time, this is beneficial in the application of electrochromic devices. Coloration efficiency (*η*), as another important parameter for EC materials, which was defined as the change in the optical density (ΔOD) for the charge consumed per unit electrode area (Δ*Q*), was calculated from the following equations:^54,55^*η* = ΔOD/Δ*Q*ΔOD = lg(*T*_b_/*T*_c_)Δ*Q* = *Q*/*A*where *T*_b_, *T*_c_ are transmittance in bleached states and colored states, respectively. *Q* was calculated from the multi-potential steps diagram, in which current is a function of time. The calculated results were tabulated in Table 2. For P1, the coloration efficiency (*η*) is 97.48 cm^2^ C^−1^ at 520 nm, 90.35 cm^2^ C^−1^ at 700 nm, 210.76 cm^2^ C^−1^ at 1550 nm. For P2, the *η* value is 142.79 cm^2^ C^−1^ at 520 nm, 87.54 cm^2^ C^−1^ at 700 nm, 283.45 cm^2^ C^−1^ at 1500 nm. While for P3, the *η* value is 143.22 cm^2^ C^−1^ at 500 nm, 85.75 cm^2^ C^−1^ at 710 nm, 304.58 cm^2^ C^−1^ at 1500 nm. Comparatively, P3 film has a higher coloration efficiency relative to the other two films.

**Table 2:** Optical contrast (Δ*T*%), response time (*t*_95%_) and coloration efficiency (*η*) of P1–P3

Copolymers	*λ*, nm	Δ*T*%	Response time(*t*_95%_), s	Coloration efficiency (*η*), cm^2^ C^−1^
P1	520	26.5	1.74	97.48
	700	10.6	0.37	90.35
	1550	55.1	0.78	210.76
P2	520	29.1	0.42	142.79
	700	19.2	0.30	87.54
	1500	43.0	0.60	283.45
P3	500	23.9	0.57	143.22
	710	21.9	0.18	85.75
	1500	67.8	1.50	304.58

**Fig. 6:**
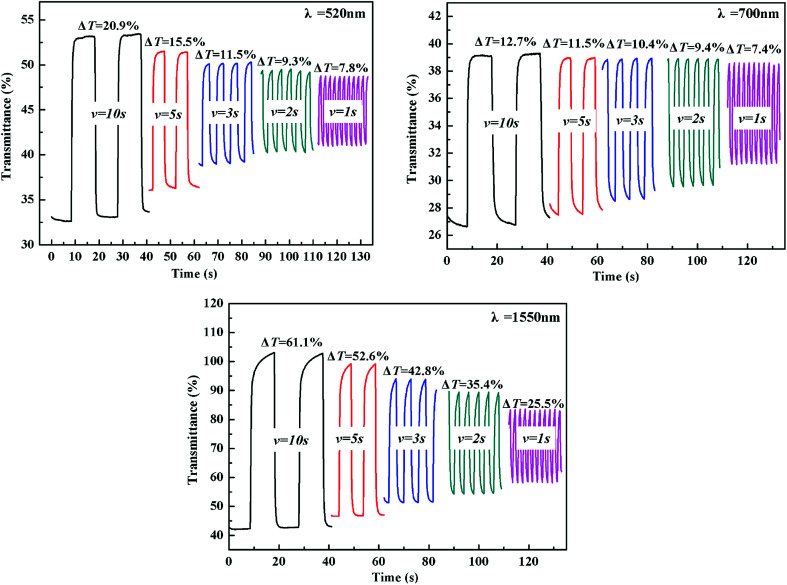
Electrochromic switching of P1 at 520 nm, 700 nm and 1550 nm with an interval of 10 s, 5 s, 3 s, 2 s, 1 s, respectively.

The electrochromic switching of the polymers with an interval of 10 s, 5 s, 3 s, 2 s and 1 s was also researched. As shown in Fig. 6, the optical contrast of P1 is 20.9% at 520 nm when switching time is 10 s but it dropped to 7.8% when switching time is 1 s with 13.1% decreased. At 700 nm, the optical contrast of P1 is 12.7% when the switching time is 10 s, but when the switching time is 1 s it dropped to 7.4% with 5.3% decreased. At 1550 nm, the optical contrast of P1 is 61.1% when switching time is 10 s, but it dropped to 25.5% when switching time is 1 s with 35.6% decreased. For P2, when the interval is changed from 10 s to 1 s, the optical contrast underwent a similar trend to that of P1, accompanied by a 2.4%, 12.3% and 4.2% decrease at 520, 710 and 1550 nm, respectively (see Fig. S10†). As for P3, there was a 6.4%, 9.7% and 21.9% decrease at 500, 710 and 1500 nm, respectively, upon the change of switching time from 10 s to 1 s (see Fig. S11†). Comparing these dates, it can be concluded that a relative bigger time interval can lead to a higher optical contrast.

### 3.7 Colorimetry

**Fig. 7:**
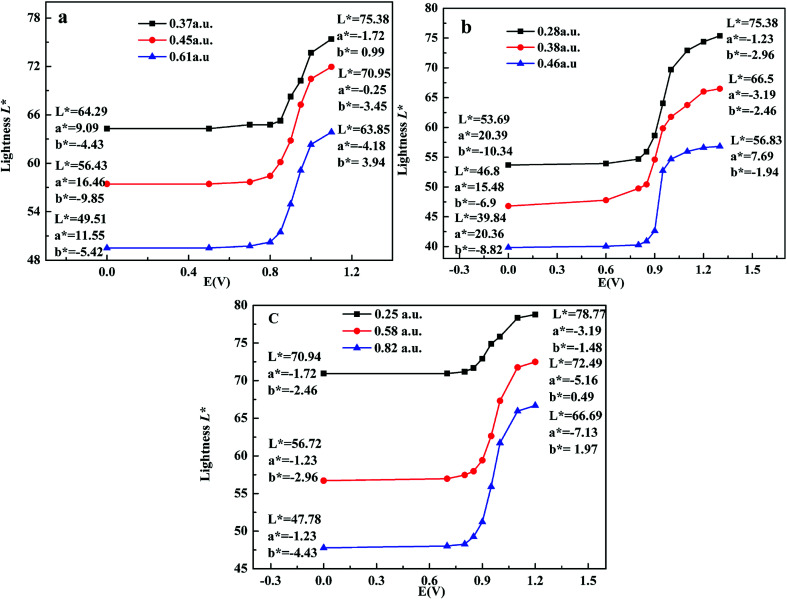
L*a*b* values of the polymers P1 (a), P2 (b) and P3 (c) with applied voltage.

Colorimetry is an important standard for judging the electrochromic devices. Here, CIE 1976 L*a*b* color space was adopted to test the color of polymers at different potentials, where L* signifies the lightness from black (0) to white (100), a* denotes the contrast between red and green, and b* means the contrast between yellow and blue.^10^ Here, the maximum absorbance was used to represent the thickness of films, as shown in Fig. 7. For P1, when the maximum absorbance increased from 0.36 a.u. to 0.50 a.u. and to 0.80 a.u., the lightness L* was 63.44, 52.52, 40.97 in neutral state and 74.41, 62.58, 59.45 in oxidized state respectively. a* means the color turned from red to green, and b* means the color turned from blue to yellow, so the color for P1 is palevioletred in neutral state and darkgray in doped state. For P2, when the maximum absorbance increased from 0.28 a.u. to 0.38 a.u. and to 0.46 a.u., the lightness L* was 70.75, 58.92, 52.84 in neutral state and 86.23, 74.19, 69.35 in oxidized state respectively, resulting in a color changed from rosybrown to silver for P2. Similarly, for P3, when the maximum absorbance increased from 0.25 a.u. to 0.58 a.u. and to 0.82 a.u., the lightness L* was 83.66, 64.52, 50.97 in neutral state and 99.35, 82.58, 66.45 in oxidized state respectively, leading to a color change from atrovirens in dedoped state to lightgrey in doped state. The above results indicate that the thinner the film is, the higher the lightness is. Therefore, the film thickness as an important parameter should be considered carefully when people want to tune the film lightness.

### 3.8 Thermal gravimetric analysis of polymers

**Fig. 8:**
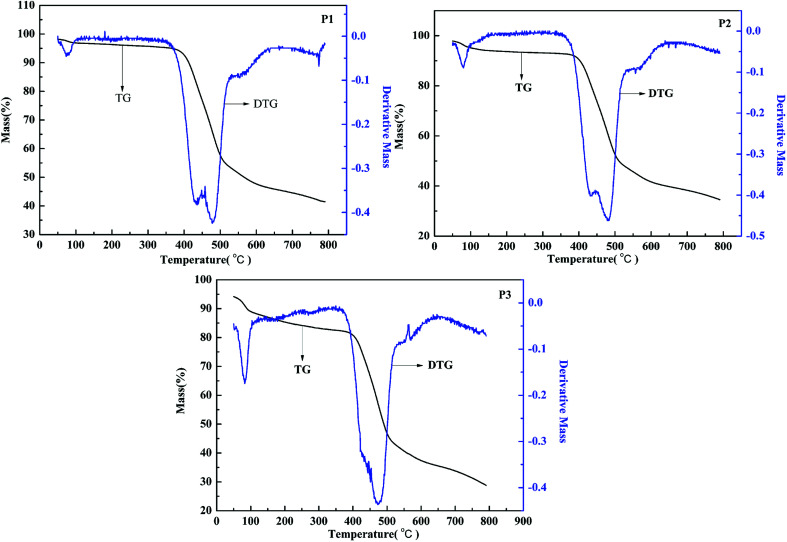
Thermal gravimetric analysis of P1–P3.

The thermostability plays an important role in electrochromic application. The thermal gravimetric (TG) analysis of the polymers was also studied, as shown in Fig. 8. The TG curve is the change of mass along with the rise of temperature, and the DTG curve is the integral of the decomposition rate. It can be seen that the decompose temperature of P1 is about 330 °C, and there exist a fast decomposition when the temperature rise from 345 °C to 478 °C. For P2, the decomposition temperature is determined to about 313 °C, followed by a fast decomposition rate from 350 °C to 480 °C. And for P3, the decomposition temperature is about 215 °C, and the polymers intensely decomposed when the temperature rise from 380 °C to 470 °C. From the above, it can be concluded that all the polymers can maintain stability when they are exposed to high temperature, which is an important quality for the electrochromic applications.

## 4. Conclusion

In conclusion, novel D–A type conjugated polymers bearing indacenodithiophene (IDT) and propylenedioxythiophene (ProDOT) units as D units and diketopyrrolopyrrole (DPP) as A units were synthesized by using Stille coupling reaction. The polymer films can be conveniently prepared *via* spray-coating method. Electrochemical and optical characteristics of the resulting polymers can be fine-tuned *via* the variation of the D–A ratios. Low level band gap (1.44 eV) and oxidation potential (1.36 V) were found for the polymer P1 with high donor content. The polymer films undergo a reversible redox process, accompanied by a dramatic color transition between the neutral state and doped state. Electrochromic switching studies indicate that the P3 film has the highest optical contrast (67.8% at 1500 nm) in NIR region, the fastest response time (0.18 s at 710 nm) and the highest coloration efficiency (304.58 cm^2^ C^−1^ at 1500 nm). Furthermore, the polymers were also found to have reasonable electrochemical stability, optical reversibility and thermostability. In light of the outstanding features above, these polymers find potential applications in electrochromic devices.

## Conflicts of interest

There are no conflicts to declare.

## Supplementary Material

RA-008-C8RA03570A-s001
