# Supplementary material for: Synthesis and characterization of novel donor–acceptor type electrochromic polymers containing diketopyrrolopyrrole as an acceptor and propylenedioxythiophene or indacenodithiophene as a donor
Source: RSC Adv. 2018 Jun 26;8(41):23119–29. doi: 10.1039/c8ra03570a (PMC9081592; doi:10.1039/c8ra03570a)
Supplement: RA-008-C8RA03570A-s001 [file RA-008-C8RA03570A-s001.pdf]

Electronic Supplementary Material (ESI) for RSC Advances.
This journal is © The Royal Society of Chemistry 2018

Supporting Information

Synthesis and characterization of novel donor-acceptor type electrochromic polymers containing diketopyrrolopyrrole as acceptor and propylenedioxythiophene or indacenodithiophene as donor

Xinfeng Cheng[a], Xiuping Ju[c], Hongmei Du[b], Yan Zhang[b], Jinsheng Zhao*[b], Yu Xie*[d]

[a]College of Chemistry and Pharmaceutical Engineering, Nanyang Normal University, Nanyang 473061, P.R. China

[b]Shandong Key laboratory of Chemical Energy Storage and Novel Cell Technology, Liaocheng University, Liaocheng, 252059, P.R. China

[c]Dongchang College, Liaocheng University, Liaocheng, 252059, P.R. China

[d]College of Environment and Chemical Engineering, Nanchang Hangkong University, Nanchang 330063, PR China.

Correspondence to: Jinsheng Zhao (E-mail: j.s.zhao@163.com); xieyu_121@163.com (Yu Xie)

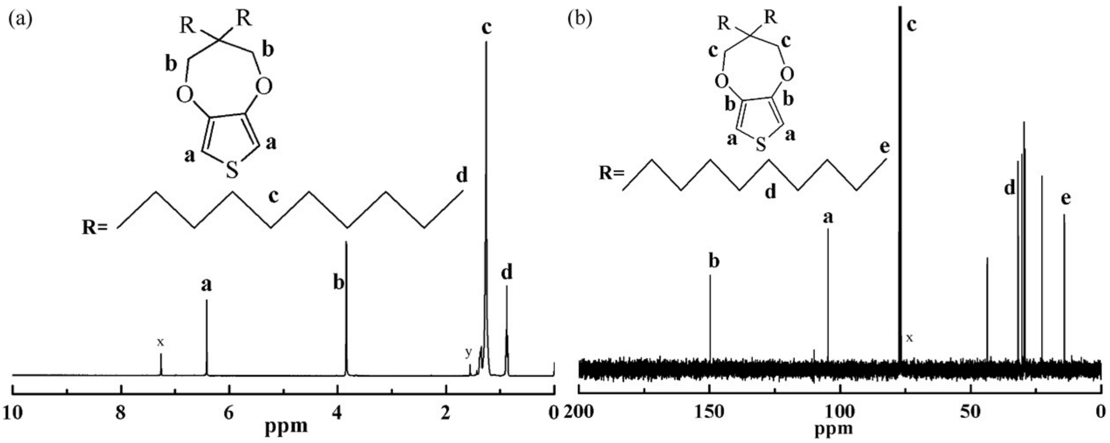

Fig. S1. ^{1}H NMR spectrum of 3,3-Bis-decyl-3,4-dihydro-2H-thieno[3,4-b][1,4]dioxepine (a), $CDCl_3$ Solvent peak and water speak were marked by 'x', 'y' respectively, ^{13}C NMR spectrum of 3,3-Bis-decyl-3,4-dihydro-2H-thieno[3,4-b][1,4]dioxepine (b), $CDCl_3$ Solvent peak were marked by 'x'.

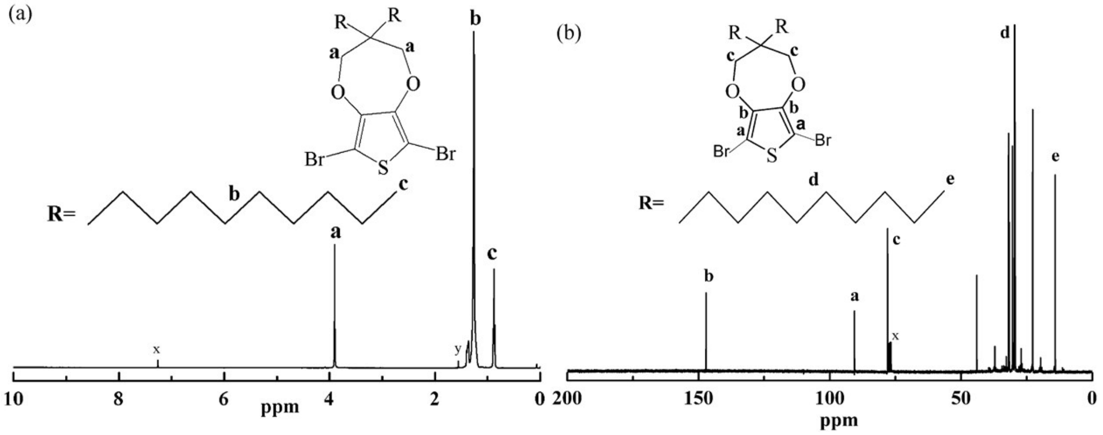

Fig. S2. ^{1}H NMR spectrum of 6,8-Dibromo-3,3-bis-decyl-3,4-dihydro-2H-thieno[3,4-b][1,4]dioxepine (a), $CDCl_3$ Solvent peak and water speak were marked by 'x', 'y' respectively, ^{13}C NMR spectrum of 6,8-Dibromo-3,3-bis-decyl-3,4-dihydro-2H-thieno[3,4-b][1,4]dioxepine (b), $CDCl_3$ Solvent peak were marked by 'x'.

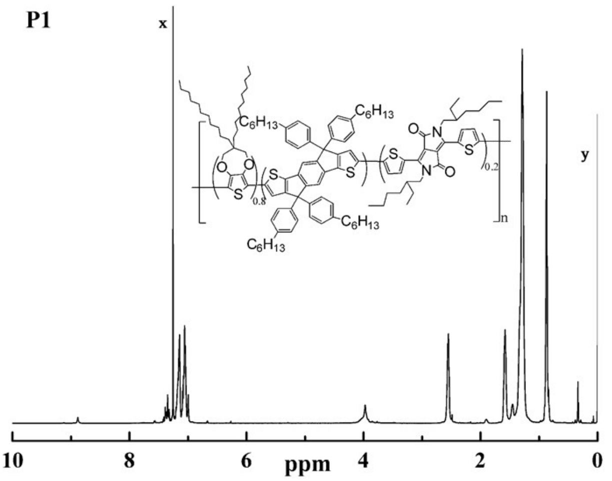

Fig. S3. ^{1}H NMR spectrum of **P1,** $CHCl_3$ Solvent and tetramethylsilane peaks were marked by ‘x’, ‘y’, respectively.

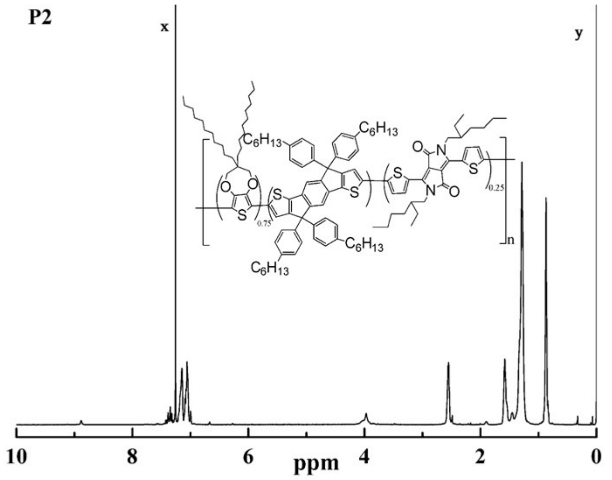

Fig. S4. ^{1}H NMR spectrum of **P2,** $CHCl_3$ Solvent and tetramethylsilane peaks were marked by ‘x’, ‘y’, respectively.

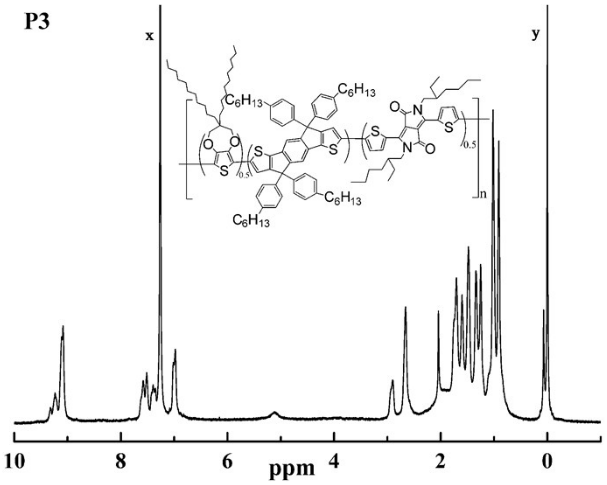

Fig. S5. ^{1}H NMR spectrum of **P3,** $CHCl_3$ Solvent and tetramethylsilane peaks were marked by ‘x’, ‘y’, respectively.

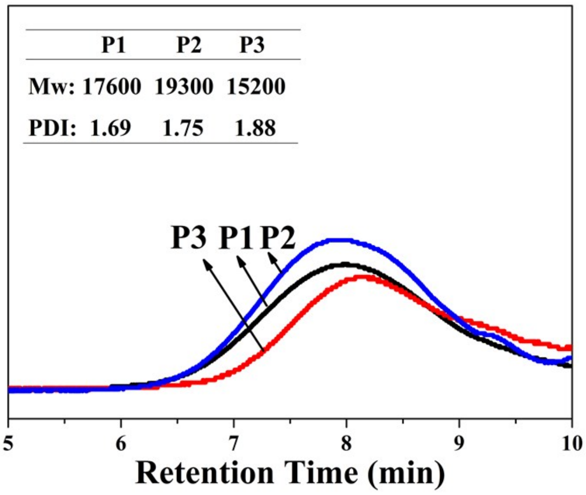

Fig. S6 GPC trace of the polymers **P1**, **P2** and **P3**.

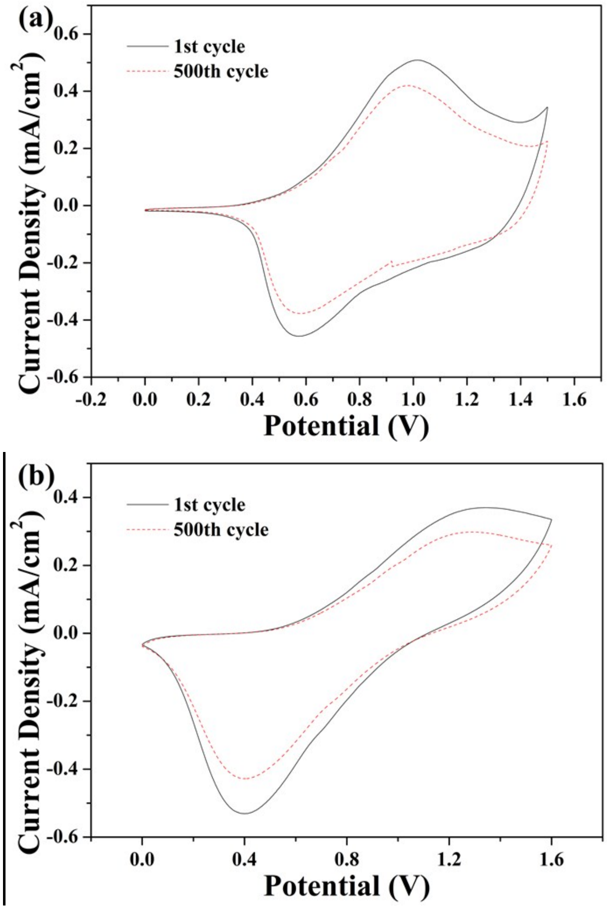

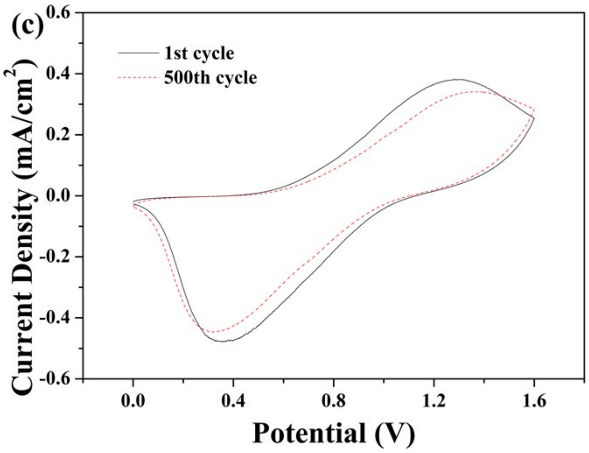

Fig. S7 CVs of the polymers **P1** (a), **P2** (b) and **P3** (c).

After 500 cycles, these polymers could retain 84%, 82% and 87% of their original electroactivity for P1, P2 and P3, respectively.

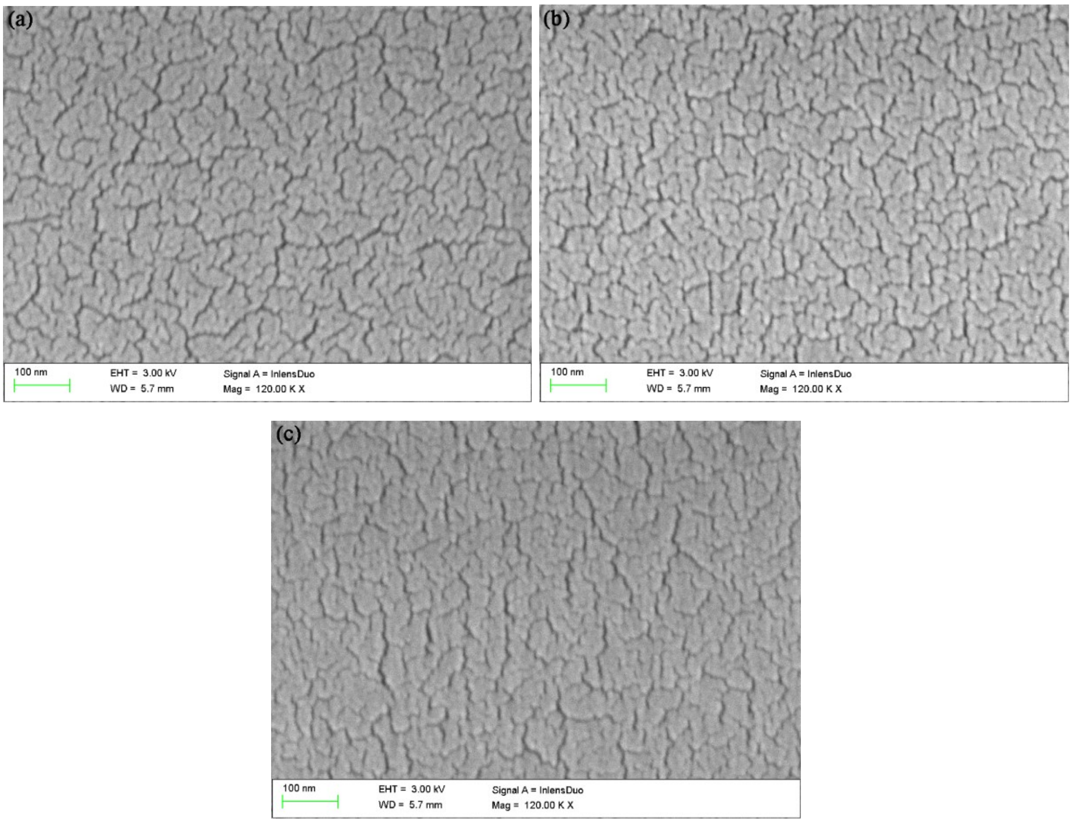

Fig. S8. SEM images of polymeric films **P1** (a), **P2** (b) and **P3** (c).

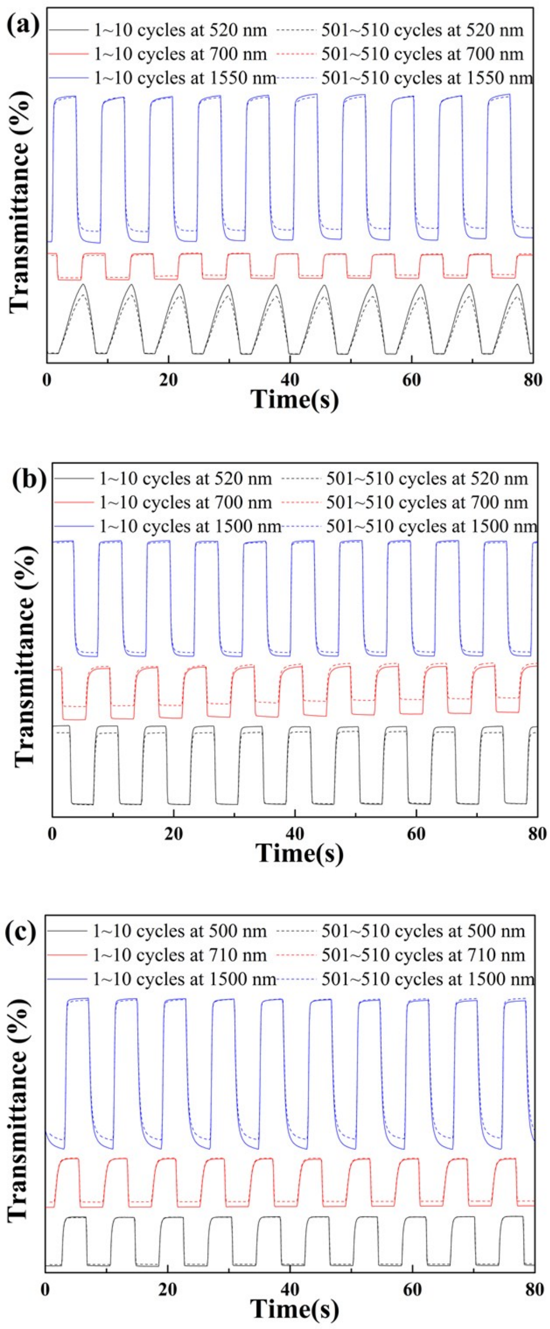

Fig. S9 Optical transmittance change of **P1**(a), **P2**(b), **P3**(c) at different wavelengths and cycle numbers.

After 500 cycles, for **P1** film, the optical contrast remained 87% at 520 nm, 88% at 700 nm and 92% at 1550 nm, respectively. For **P2** film, the optical contrast remained 91% at 520 nm, 78% at 700 nm and 95% at 1500 nm after 500 cycles, respectively. While for **P3** film, the optical contrast remained 96% at 500 nm, 91% at 710 nm and 93% at 1500 nm after 500 cycles, respectively.

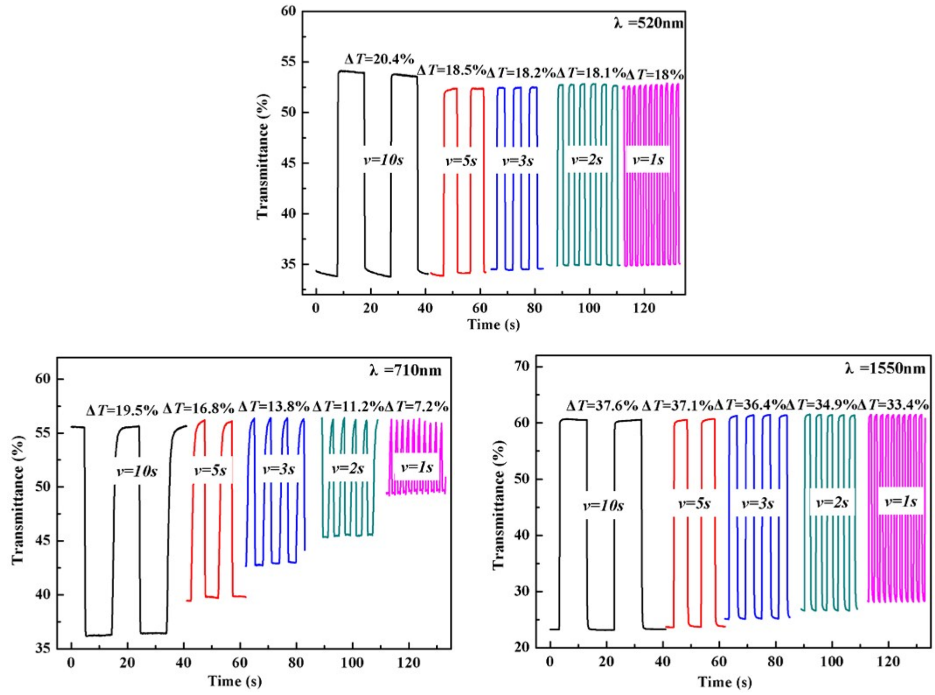

Fig. S10. Electrochromic switching of **P2** at 520 nm, 710 nm and 1550 nm with an interval of 10 s, 5 s, 3 s, 2 s, 1s, respectively.

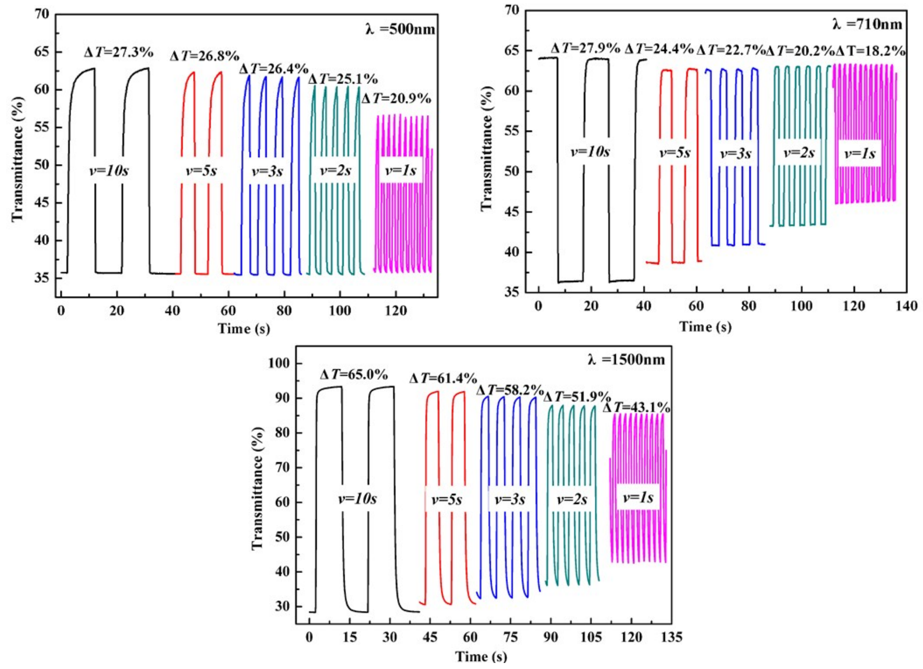

Fig. S11. Electrochromic switching of **P3** at 500 nm, 710 nm and 1550 nm with an interval of 10 s, 5 s, 3 s, 2 s, 1s, respectively.
